# Ecotourism and the Hidden Ecology of Infection: The Andes Virus Cruise Outbreak

**DOI:** 10.3390/idr18030059

**Published:** 2026-06-16

**Authors:** Laura Scorzolini, Alessandra D’Abramo, Enrico Girardi, Emanuele Nicastri

**Affiliations:** 1 Clinical and Research Infectious Diseases Department, National Institute for Infectious Diseases Lazzaro Spallanzani-IRCCS, Via Portuense 292, 00149 Rome, Italy; laura.scorzolini@inmi.it (L.S.); emanuele.nicastri@inmi.it (E.N.); 2 Scientific Direction, National Institute for Infectious Diseases Lazzaro Spallanzani-IRCCS, Via Portuense 292, 00149 Rome, Italy; enrico.girardi@inmi.it

## 1. Background

Over the past decade, outdoor recreational activities—including ecotourism, wildlife observation, adventure travel, and expedition cruising—have expanded at an unprecedented pace. While widely praised for their environmental, educational, and economic benefits, these activities increasingly place travellers in ecosystems where zoonotic pathogens are endemic. The intersection of human mobility, wildlife exposure, and delayed clinical recognition has emerged as a critical but under-addressed driver of global infectious disease risk [1]. The 2026 multinational outbreak aboard a Dutch cruise ship illustrates how a single environmental exposure in an endemic region—most plausibly through inhalation of aerosolized rodent excreta in Argentina prior to embarkation—can initiate a chain of events extending far beyond its ecological origin. Once introduced onto the ship, transmission dynamics shifted. Although environmental exposure was the primary trigger, subsequent limited person-to-person spread—consistent with the unique transmission properties of ANDV—likely occurred in the confined setting of the vessel. This outbreak therefore highlights the dual mechanism through which ecotourism-associated infections can propagate: initial zoonotic spillover followed by amplification in high-density environments such as cruise ships. Importantly, it underscores that such settings function not as primary sources of infection but as facilitators of secondary transmission. Subsequent limited human-to-human transmission in close-contact settings likely amplified the outbreak on board, highlighting the role of confined environments as facilitators rather than primary drivers of transmission. This event shows how leisure travel can overwhelm international surveillance and reveal gaps in the current One Health framework [2]. The cruise outbreak exemplifies how these trends translate into epidemiological risk; travel to remote areas with limited surveillance, close proximity to rodent habitats, and activities that increase exposure to contaminated aerosols all contributed to infection risk prior to embarkation [3,4]. Hantaviruses are rodent-borne RNA viruses, recognized as an emergent zoonotic infection with a global distribution. Hantaviruses belong to the *Orthohantavirus* genus, family *Hantaviridae*, order *Bunyavirales* [5]. Two serious diseases in humans were recognized: hemorrhagic fever with renal syndrome (HFRS) (Europe and Asia), and hantavirus cardiopulmonary syndrome (HCPS) (Americas). Case fatality rate (CFR) varies across Hantavirus infections, ranging from 0.1 to 12% in HFRS disease and from 30 to 40% in HCPS, respectively [6,7]. Hantavirus is primarily contracted through environmental exposure to infected rodents, which serve as reservoirs. Transmission occurs mainly through the inhalation of aerosolized viral particles present in the biological fluids of rodents. There is no evidence of human-to-human transmission of HFRS causing hantaviruses, although blood products drawn from infected individuals or blood transmission during medical interventions are a possible source of infection [8]. Person-to-person transmission of ANDV has been documented in Argentina and Chile [9,10]. Within this context, ANDV is particularly relevant. Unlike most hantaviruses, it is the only species with well-documented human-to-human transmission, typically requiring close and prolonged (i.e., contact with a sexual partner, tongue kissing, sleeping in the same room, or attending a social gathering with a symptomatic person). Its prolonged and variable incubation period (up to 4 days and 42 days) complicates timely diagnosis and facilitates undetected cross-border spread [9,11]. These characteristics make ANDV uniquely suited to exploit travel-associated networks, as demonstrated by the cruise ship outbreak, where a single exposure event led to multiple cases, including fatalities, across geographically dispersed regions [12].

The outbreak aboard the MV Hondius (April–May 2026) represents a first clearly documented transmission cluster in a maritime transport setting. This outbreak serves as a paradigmatic example of how ecotourism can drive emerging infectious disease risks: a localized zoonotic exposure, followed by limited human-to-human transmission in a closed setting, culminating in widespread international dissemination [13].

## 2. Practical Aspects for Clinicians and Public Health

Outdoor recreational activities increasingly function as sentinel events for zoonotic spillover, highlighting how specific behavioural exposures—rather than geography alone—can precipitate infection. Greater awareness of these risk factors is therefore essential to prevent severe and potentially fatal outcomes. The recent outbreak further illustrates that ANDV, despite its relatively limited transmissibility, can still trigger complex international public health crises. In this context, expedition travel platforms—particularly cruise ships—act not as primary sources of infection but as epidemiological amplifiers, facilitating secondary transmission through prolonged close contact among travellers.

## 3. Broader Implications for One Health and Travel Medicine

These observations reinforce the need to strengthen One Health-integrated surveillance systems, capable of linking environmental, animal, and human health data to enable early detection and response. At the same time, travel medicine must evolve beyond its traditional focus on vaccines and prophylaxis for vector-borne diseases, incorporating eco-epidemiological literacy to better prepare both clinicians and travellers for the risks associated with increasing human interaction with natural ecosystems. Current systems remain predominantly reactive and clinically centred, despite growing evidence that environmental and behavioural exposure data are essential for early warning [4]. Nowadays, there is no structured pre-travel zoonotic risk counselling for ecotourists; in addition, limited integration of wildlife and environmental surveillance into travel medicine conveys an inconsistent post-travel follow-up for asymptomatic travellers from endemic regions. The documented ANDV person-to-person transmission in Argentina underscores the urgent need for standardized protocols for early case recognition, monitoring, and management of exposed individuals. The prompt identification of exposed contacts remains critical, given the virus’s prolonged incubation period and the potential for secondary transmission within households and other close-contact settings [14].

## 4. Clinical Research Priorities

Comprehensive genomic surveillance of Andes virus (ANDV) in both human and animal populations is essential, ideally implemented within a One Health framework that integrates human, animal, and environmental health domains. The incorporation of multi-omics approaches (genomics, transcriptomics, proteomics, and metabolomics), combined with network medicine methodologies, is needed to elucidate the complex biological interactions underlying viral transmission dynamics and disease pathogenesis. Currently, no specific antiviral, immunomodulatory therapy, or vaccines are approved. Early post-exposure therapeutic strategies, particularly among individuals at the highest risk of infection, have to be evaluated and warranted. Candidate interventions include favipiravir, due to already promising data for safety/PK (pharmacokinetic) and dose selection, which might be administered during the pre-viraemic phase. Ribavirin has not been shown to be effective in the treatment of HCPS [15]. To date, there is no direct experimental or clinical evidence supporting the efficacy of molnupiravir in hantavirus infection, including Andes virus [16]. Promising antivirals, including baloxavir, as well as entry inhibitors such as homodimeric lectins and coumarin derivatives with antiviral activity, are currently in preclinical studies. Icatibant, an immunomodulatory bradykinin B2 receptor antagonist, has shown improved outcomes in antagonizing vascular leakage. Therefore, further data are needed to evaluate its possible effectiveness, and its use in severe cases should be considered [17]. Passive immunotherapy using monoclonal or neutralizing antibodies showing antiviral activity in in vitro studies needs to be progressed to clinical testing, including the option of combination therapy or PEP if they show the long half-life features [17]. Further studies to confirm the safety and efficacy of hyperimmune convalescent plasma are required. Active vaccination, including conventional and mRNA/DNA vaccines, may also have a role, as seen in rabies, given the prolonged incubation period. All these single or combined approaches may be especially relevant for close household contacts of confirmed ANDV cases, individuals exposed following high-risk encounters, laboratory accidents, during super spreader events, or during cold-water cruise ship outbreaks, where the likelihood of transmission is amplified. Finally, primary immunoprophylaxis strategies—including conventional and mRNA vaccines—for residents of endemic areas are under development but remain far from clinical implementation [17].

## 5. Systemic Challenges Regarding Global Surveillance

The current ANDV outbreak placed extraordinary pressure on global surveillance and response mechanisms, revealing several systemic challenges:

1. The limited awareness of rare zoonoses in non-endemic settings underscores the need to systematically integrate travel history into clinical assessment and to strengthen the connection between travel medicine and frontline care in order to reduce the risk of delayed diagnosis
2. Existing interoperable surveillance systems and standardized cross-border data-sharing mechanisms, as were well documented during the COVID-19 pandemic and during the West African Ebola outbreak, highlight the critical importance of coordinated approaches and efficient data exchange in managing complex multinational contact tracing.
3. Targeted post-travel surveillance, including active follow-up and serological studies, is needed to better define the true burden of imported zoonotic infection, including asymptomatic and mild infections.
4. Environmental investigations, including rodent surveillance, highlight the value of integrating ecological monitoring into outbreak response within a One Health framework, particularly in tourism-dependent areas.

## 6. Conclusions

The Andes virus outbreak linked to expedition cruising in South America is not an anomaly but a signal event. It illustrates how ecotourism and global mobility can convert localized zoonotic exposures into transnational public health challenges, revealing gaps in current surveillance and response systems.

This episode provides an opportunity to strengthen existing approaches by more systematically integrating travel medicine into surveillance strategies. Enhancing pre-travel risk assessment, developing structured post-travel follow-up for high-risk exposures, and reinforcing coordination between clinical and public health systems could improve early detection and response.

Embedding these elements within a One Health framework, alongside greater attention to ecotourism-related risks, would support more resilient surveillance systems and help mitigate the impact of similar events in the future.

## Data Availability

All relevant data are within the manuscript.

## Abbreviations

The following abbreviations are used in this manuscript:

ANDV	Andes virus
CFR	Case fatality rate
DOBRV	Dobrava virus
HCPS	Three-letter acronym
HFRS	Hemorrhagic fever with renal syndrome
IHR	International Health Regulation
PEP	Post-exposure prophylaxis
PUUV	Puumala virus
PK	Pharmacokinetic
SNV	Sin Nombre virus
